# Accelerated Reendothelialization, Increased Neovascularization and Erythrocyte Extravasation after Arterial Injury in BAMBI^−/−^ Mice

**DOI:** 10.1371/journal.pone.0058550

**Published:** 2013-03-01

**Authors:** Nicolas Guillot, Dmitrij Kollins, Juan J. Badimon, Detlef Schlondorff, Randolph Hutter

**Affiliations:** 1 Department of Medicine, Mount Sinai School of Medicine, New York, New York, United States of America; 2 Cardiovascular Institute, Mount Sinai School of Medicine, New York, New York, United States of America; Osaka University Graduate School of Medicine, Japan

## Abstract

**Background:**

Intimal injury rapidly activates TGFβ and enhances vascular repair by the growth of endothelial (EC) and vascular smooth muscle cells (VSMC). The response to the TGFβ family of growth factors can be modified by BAMBI (BMP, Activin, Membrane Bound Inhibitor) acting as a non-signaling, competitive antagonist of TGFβ type I receptors such as ALK 1 and 5. In vivo the effect of BAMBI will depend on its cell-specific expression and of that of the ALK type receptors. We recently reported EC restricted BAMBI expression and genetic elimination of BAMBI resulting in an in vitro and in vivo phenotype characterized by endothelial activation and proliferation involving alternative pathway activation by TGFβ through ALK 1.

**Methodology/Principal Findings:**

To test the hypothesis that BAMBI modulates arterial response to injury via its effects on endothelial repair and arterial wall neovascularization we used a model of femoral arterial denudation injury in wild type (WT) and BAMBI^−/−^ mice. Arterial response was evaluated at 2 and 4 weeks after luminal endothelial denudation of femoral arteries. The BAMBI^−/−^ genotype mice showed accelerated luminal endothelial repair at 2 weeks and a highly unusual increase in arterial wall neovascularization compared to WT mice. The exuberant intimal and medial neovessel formation with BAMBI^−/−^ genotype was also associated with significant red blood cell extravasation. The bleeding into the neointima at 2 weeks transiently increased it’s area in the BAMBI^−/−^genotype despite the faster luminal endothelial repair in this group. Vascular smooth muscle cells were decreased at 2 weeks in BAMBI^−/−^ mice, but comparable to wild type at 4 weeks.

**Conclusions/Significance:**

The absence of BAMBI results in a highly unusual surge in arterial wall neovascularization that surprisingly mimiks features of intra-plaque hemorrhage of advanced atheroma in a mechanical injury model. This suggests important effects of BAMBI on arterial EC homeostasis that need to be further studied in a model of inflammatory atherosclerosis.

## Introduction

Arterial injury induces a rapid activation of Transforming Growth Factor β (TGFβ) signaling in the arterial wall. TGFβ signaling regulates vascular repair via the modulation of endothelial cell (EC) and vascular smooth muscle cell (VSMC) migration and proliferation [1]. TGFβ overexpression has been shown to enhance neointimal hyperplasia (IH), whereas inhibiting TGFβ can attenuate neointimal growth [2], [3]. In EC TGFβ modulation takes place predominantly through ALK-1 and non-canonical pathways to promote proliferation [4], while in VSMC, TGFβ acts likely via ALK-5 signaling [4]–[6]. The novel molecule BAMBI (BMP, Activin, Membrane Bound Inhibitor) in turn modifies the cellular response to the TGFβ family of growth factors by acting as a non-signaling, competitive antagonist of TGFβ type I receptors such as ALK 1 and 5 [7], [8]. The specific effect of BAMBI on TGFβ signaling is dependent on the combination of TGFβ receptor and BAMBI expression on cells. EC express specifically ALK 1 and we recently reported EC restricted BAMBI expression [9]. Consistent with the coexpression of ALK 1 and BAMBI in endothelial cells, genetic elimination of BAMBI resulted in a phenotype characterized by *in vitro* and *in vivo* endothelial activation and proliferation [9]. We also showed that in EC this phenotype could be mechanistically attributed to enhanced alternative TGFβ signaling through ALK 1 and ERK2 and SMAD 1/5 activation after BAMBI elimination [9]. To explore the effect of BAMBI on arterial response to injury we performed femoral arterial (FA) endothelial denudating injury [10], [11] in wild-type (WT) and BAMBI^−/−^ mice. The effects of BAMBI on re-endothelialization, arterial wall neovascularization and neointima formation were consistent with BAMBI playing an important role in endothelial homeostasis and repair.

## Methods

### Ethics Statement

All animal studies were carried out with compliance with the Mount Sinai School of Medicine Institutional Animal Care and Use Committee approved protocols (protocol number LA08-00399).

### Animals

BAMBI^−/−^ mice, on C57BL/6 background, were generated as reported [12]. All experiments were conducted in accordance with the guidelines of the US National Institutes of health and were approved by the Institutional Animal Care and Use Committee at the Mount Sinai School of Medicine.

### Femoral Artery Injury

Mice (male, 7–12 week-old) were anesthetized with isoflurane (Baxter healthcare Corp., IL, USA). Transluminal injury to the common femoral artery was produced by 3 passages of a 0.25 mm diameter angioplasty guidewire (SilverSpeed-10, Micro therapeutics, Inc, CA, USA) as detailed and validated by Roque et al. (2000) [13].

### Histology and Immunohistochemistry

Animal were euthanized 2 and 4 weeks after arterial injury and perfusion-fixed with 4% paraformaldehyde in PBS (pH 7.4). Hindlimbs were excised en bloc and fixed overnight in formalin then decalcified in 1% formic acid [10], [11], [13]. The femoral artery was embedded in paraffin and subsequently serially sectioned (4 µm thick) from the distal site. The serial sections were then immunostained with antibodies against biotynylated - Griffonia simplicifolia isolectin B4, (10 µg/mL, Vector laboratories, CA, USA) and confirmed by the use of von Willebrand factor antibody (1∶75, Abcam, MA, USA) and counterstained with hematoxylin. High power light microscopic images (×400) of these immunostainings, showing the whole circumference of the injured femoral artery, were digitally acquired and evaluated by computer-aided morphometry.

The degree of luminal endothelial cell coverage was determined by tracing and measuring the length of segments of luminal circumference covered with either isolectin B4 or vWF immunostaining and to add those segments to a combined length for each arterial specimen. The percentage of re-endothelialization reflected the ratio between the combined length of luminal endothelial immunostaining and total luminal circumference. A mean value of luminal endothelial cell coverage was derived for each artery from the morphometric analysis of 2 tissue sections. For staining of smooth muscle cells, α-actin (αSMA) antibody was used (alkaline phosphatase conjugated monoclonal anti-α-smooth muscle actin, clone 1A4, 1∶100, Sigma, MO, USA). Endothelial cells proliferation was evaluated by Ki67 staining (1∶500, Vector laboratories, CA, USA). Staining was carried out according to the manufacturer’s protocol followed by the horseradish-peroxydase-conjugated streptavidin technique and developed with 3,3′- diaminobenzidine (Vector laboratories, CA, USA). Sections were counterstained with hematoxylin. Appropriate negative controls for all of the immunological stains were performed (Figure S1). All of these methods have been reported previously by our group [10], [11].

Fibrin, extracellular matrix and erythrocyte content were defined using Carstairs’ method [14]. Paraffin sections were hydrated and placed in mordant 5% ferric ammonium sulfate for 5 min rinsed and stained with Mayer’s hematoxylin for 5 min and rinsed under tap water. Sections were then stained with picric acid-orange G solution for 1 hour and stained in Ponceau-fuchsin solution for 5 minutes. After being rinsed sections were differentiated with 1% phosphotungstic acid and rinsed in distilled water. Sections were eventually stained with aniline blue solution for 15 min then rinsed, dehydrated, cleared and mounted. Fibrin appears red-orange to bright red, collagen bright blue, red blood cells yellow, platelets gray-blue to navy and muscle red.

### Morphometry

Morphometric analysis was performed using National Institutes of Health (NIH) ImageJ version 1.43 software: area of the lumen, intima, media and vessel, lengths of the internal elastic lamina (IEL) and external elastic lamina (EEL) and the intima-to-media (I/M) ratio were calculated as previously described [13], [15]. Arteries with occlusive thrombus formation and/or disrupted elastica interna were excluded from the analysis.

### Statistical Analysis

Data are presented as mean ± SEM. For all assays, statistical analysis was performed using a Mann-Whitney U test. Results were considered significant if P<0.05.

## Results and Discussion

### Accelerated Reendothelialization and Increased Arterial Wall Neovascularization after Arterial Injury with BAMBI^−/−^ Genotype

**Figure 1:**
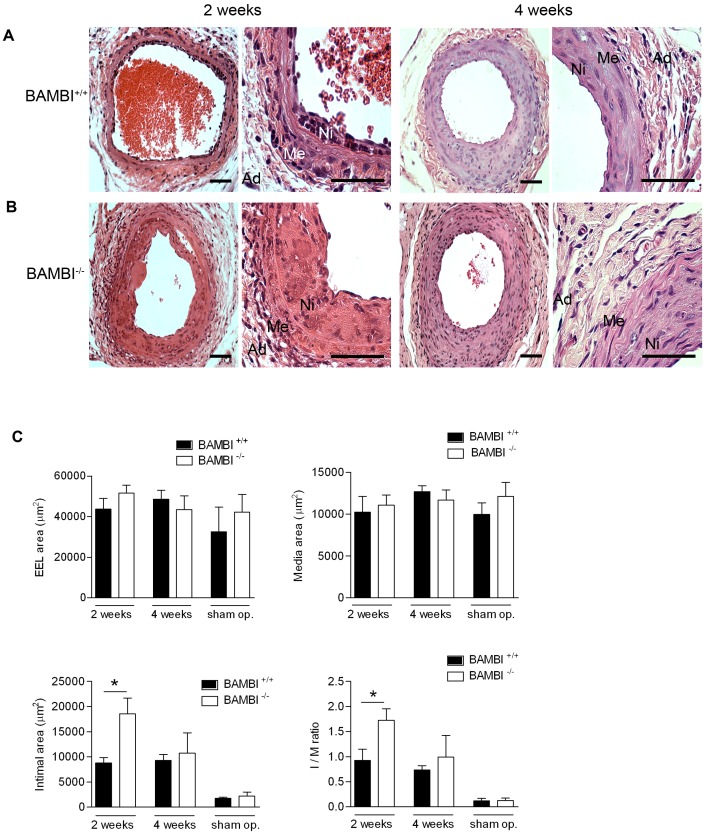
Deficiency of BAMBI increases early neo-intimal hyperplasia after intima denudation. Representative pictures of femoral arteries from BAMBI^+/+^ (**A**) and BAMBI^−/−^ (**B**) mice at 2 and 4 weeks after injury. (Hematoxylin and eosin stains. Original magnification ×400 and ×1000; bar = 50 µm; Ni: neointima, Me: media and Ad: adventitia). (**C**). Morphometric analysis of the femoral arteries. Data are mean ± SEM; n = 5–9; *P<0.05 compared to respective wild type.

**Figure 2:**
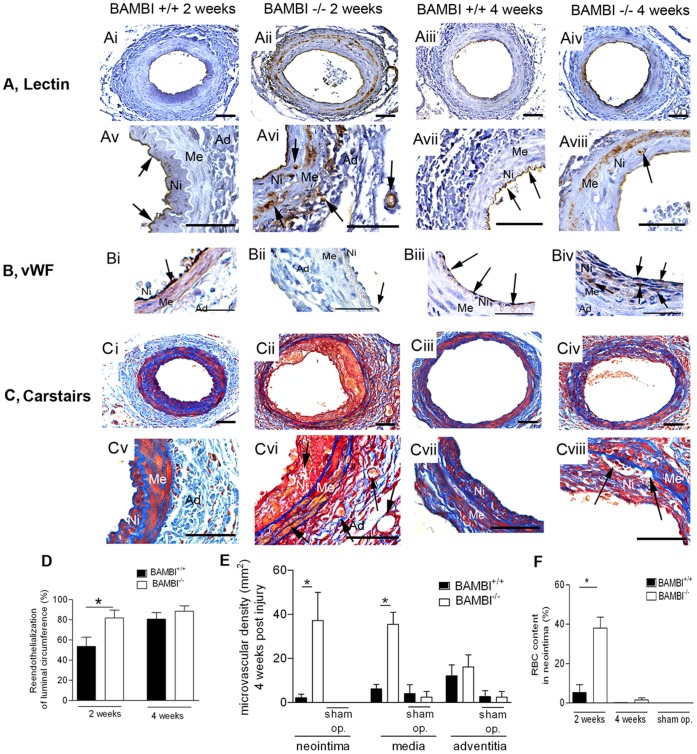
BAMBI deficiency accelerates reendothelialization and increased neo-intimal neovascularization and accumulation of erythrocytes. (**A**) Endothelial cells of femoral arteries were stained by isolectin B4 in BAMBI^+/+^ and BAMBI^−/−^ mice 2 and 4 weeks after intimal denudation. (Ni: neointima, Me: media and Ad: adventitia. Black arrows indicate lectin positive luminal endothelial cells and intimal microvessels, original magnification ×400 upper panel and ×1000 lower panel; bar = 50 µm). (**B**) Reendothelialization was also determined by staining with von Willebrand factor (black arrows) magnification ×1000.(**C**) Fibrin and erythrocytes were visualized by Carstairs’ staining of femoral arteries at the same time point (Original magnification ×400 upper panel and ×1000 lower panel; bar = 50 µm; black arrows indicate RBC infiltration). (**D**) Quantification of the reendothelialization of the femoral arteries. (**E**) Microvascular density in the different layers of the femoral arteries 4 weeks after injury. (**F**) Erythrocytes accumulation in neointima 2 and 4 weeks after injury. Data are mean ± SEM, n = 5–9, *P<0.05 compared to respective wild type.

**Figure 3:**
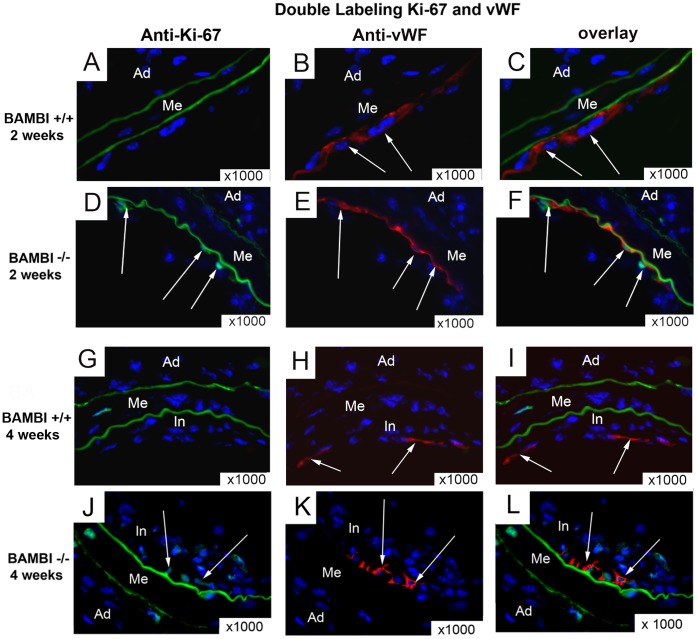
BAMBI deficiency results in neo-intimal neovasculariztion with proliferating endothelial cells. Proliferation of endothelial cells was evaluated by double staining for the proliferation marker Ki67 (green) and the endothelial cell marker vWF (red); co-localization of both signals showing as yellow (overlay) The lamina elastica interna shows typical green autofluorescence. At two weeks double positive cells (white arrows) were restricted to the luminal surface in the BAMBI^−/−^ mice, whereas at 4 weeks double positive cells also appeared deep in the neointima in a pattern consistent with ongoing neo-vessel formation (original magnification ×1000).

Histo-morphometric analysis of arteries at 2 and 4 weeks after endothelial denudation revealed increased neointima formation without a change in media or total vessel size in the BAMBI^−/−^ compared to WT mice; resulting in a significantly greater intima/media ratio at 2 weeks (Figure 1A–C). Staining for BAMBI in WT mice confirmed its presence restricted to endothelial cells and also showed, that all regenerating endothelial cells at two weeks after denudation expressed BAMBI (Figure S2). Reendothelialization was significantly accelerated with BAMBI^−/−^ genotype as measured by morphometric evaluation of isolectin staining showing 79% of the luminal circumference covered by endothelial cells at 2 weeks after injury in BAMBI^−/−^ mice compared to 52% in WT mice (*P*<0.05) (Figure 2A and D). Comparable results were obtained with staining of endothelial cells by von Willebrand factor (Figure 2B). By morphometric quantification 71.4±6.7% of luminal circumference were covered at 2 weeks in BAMBI^−/−^ mice (n = 6 ) versus 52±3% in wild type mice (n = 5; P<0.04). At 4 weeks, reendothelialization was almost complete in both groups, no longer showing a significant difference with 92% in BAMBI^−/−^ and 84% in WT mice (Figure 2A, B and D, *P* = NS). Enhanced proliferation of endothelial cells contributed to the accelerated reendothelialization in the BAMBI^−/−^ mice at 2 weeks as evaluated by KI-67 staining for proliferating luminal cells (7.7×10^−3^±1.6 KI-67 positive cells per micrometer of luminal surface in BAMBI^+/+^ mice versus 15.9×10^−3^±2.7 in BAMBI^−/−^ mice; n = 4–6 per group; *P*<0.04). At 4 weeks after denudation the number of KI67 positive luminal cells was no longer significantly different between BAMBI^+/+^ and BAMBI^−/−^ mice (not shown). The endothelial nature of the KI-67 positive luminal cells was confirmed by double staining with vWF (Figure 3). Of note, enhanced endothelial growth was not limited to macro-vascular endothelial cells repairing the luminal surface. We also observed neovascularization in the media and neointima with BAMBI^−/−^ genotype, two compartments of the arterial wall where neovascularization is normally minimal or undetectable in this model as demonstrated by the absence of neovessels in the WT mice after endothelial denudation (Figure 2A and B and quantified in Fig. 2E). The contribution of proliferating endothelial cells to neo-vessel formation in the neo-intima at 4 weeks is illustrated by the presence of KI67/vWF double positive endothelial cells within the neointima of BAMBI^−/−^ mice (Figure 3). These findings are consistent with an activated EC phenotype in BAMBI^−/−^ mice, and would support the hypothesis of an inhibitory function of BAMBI on EC proliferation in response to TGFβ through ALK 1 and alternative signaling, as recently reported by us [9]. Thus the accelerated reendothelialization and marked neovascularization in BAMBI^−/−^ mice could potentially be explained by the enhanced alternative pathway response to the TGFβ generated at the site of vascular injury [3], [16]–[18].

### Unusual Erythrocyte Extravasation during Early Neointima Formation

Surprisingly, the more rapid reendothelialization process in BAMBI^−/−^ mice was associated with a larger neointima at 2 weeks, whereas it is generally observed, that more rapid reendothelialization results in less intima hyperplasia [19]. Therefore we further examined the neointima composition using Carstairs’ staining for identification of erythrocytes, collagen, and fibrin [14]. At two weeks the accumulation and extravasation of erythrocytes was abundant in the neointima of BAMBI^−/−^ mice while completely absent in the neointima of WT mice (*P*<0.05) (Figure 2C and F). The erythrocyte extravasation in BAMBI^−/−^ mice was also apparent by H&E staining (Figure 1A and B). At 2 weeks erythrocyte accumulation accounted for nearly 40% of the thickened neointimal area of BAMBI^−/−^ mice. At 4 weeks, most erythrocytes had disappeared, presumably by lysis and phagocytosis, and the neointima had been converted to its typical fibrotic phenotype (Figure 1A and B and Figure 2C and F). These observations suggest leakiness and immaturity of the newly formed microvessels in the BAMBI^−/−^ mice probably due to the activated endothelial phenotype reported by us [9]. The observation that a faster reendothelialization process was associated with enlarged neointima at 2 weeks in BAMBI^−/−^ mice may seem surprising since previous reports linked more rapid reendothelialization to less intimal hyperplasia [19]. However, the early increase in neointima area in the BAMBI^−/−^ mice is not attributable to increased VSMC’s but rather to the extensive and highly unusual “bleed in” phenomenon (Figure 2, C and F). At 4 weeks, and after disappearance of the erythrocyte material, no significant difference in neointima area was observed between the two groups (Figure 2F).

### Delayed VSMC Infiltration in Neointima from BAMBI^−/−^ Mice

**Figure 4:**
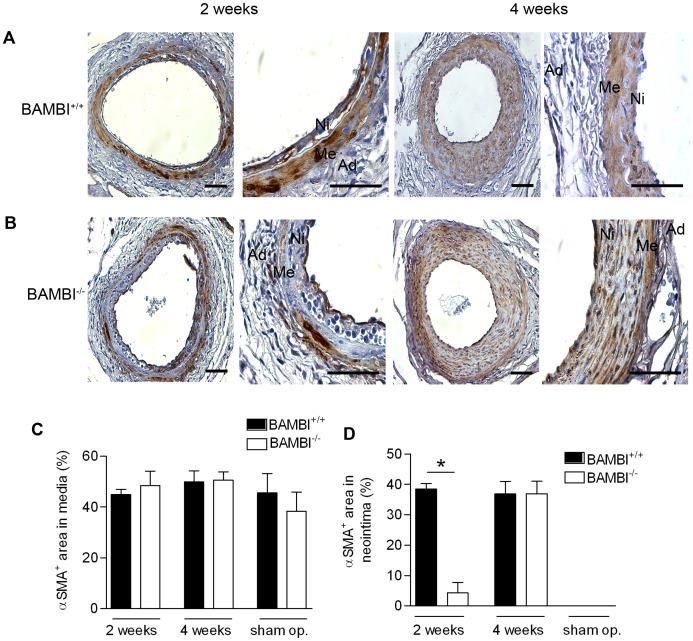
Smooth muscle cells in neointima and media from BAMBI^+/+^ and BAMBI ^−/−^ mice. Staining for alphaSMA in the femoral arteries from BAMBI^+/+^ (A) and BAMBI^−/−^ (B) mice 2 and 4 weeks after injury. (Original magnification ×400 and ×1000; bar = 50 µm; Ni: neointima, Me: media and Ad: adventitia). (C) and (D) alphaSMA positive areas in media and neointima of the femoral arteries. Data are mean ± SEM, n = 5–9, *P<0.05 compared to respective wild type.

VSMC contribute to the neointima and arterial wall remodeling after vascular injury, and we therefore examined the VSMC content using αSMA staining (Figure 4A–C). VSMC content of the media was comparable between BAMBI^+/+^ and BAMBI^−/−^ mice after 2 and 4 weeks (Figure 4A–C). In contrast the relative VSMC area of the neointima was reduced in the BAMBI^−/−^ mice at 2 weeks (Figure 4A, B and D). However this can at least in part be explained by the marked increase in neointimal area due to the blood extravasation rather than to a decrease in absolute VSMC area. Consistent with this interpretation VSMC areas were comparable in neointima from wild type and BAMBI^−/−^ mice at 4 weeks, a time point at which the neointimal blood extravasation had been largely resorbed (Figure 3). The absence of VSMC expansion in the neointima of BAMBI^−/−^ mice is surprising as it occurred in spite of the extravasation, which is considered to enhance neointimal VSMC immigration and proliferation [20].

Based on these and our previous results [9] we hypothesize that BAMBI elimination results in different TGFβ signaling and effects in EC and VSMC: in EC, BAMBI elimination enhances TGFβ effects via ALK-1 and alternative pathway activation resulting in proliferation and neo-angiogenesis, while in VSMC BAMBI elimination would enhance ALK-5 and canonical pathway signaling and inhibit VSMC proliferation [8], [21]–[23]. During the initial phase of reendothelilization this could result in an unstable neointima with intra-intimal bleeding, while at later stages marked neo-angiogenesis occurs, as noted in the BAMBI^−/−^ mice.

In summary genetic elimination of BAMBI resulted in accelerated endothelial repair of the luminal arterial surface after denudating injury. BAMBI^−/−^ genotype was also associated with strikingly increased arterial wall neovascularization and a highly unusual extravasation and accumulation of erythrocytes into the intimal space. These findings in a mechanical injury model surprisingly mimic features of intra-plaque hemorrhage of advanced atheroma in atherosclerosis. Thus BAMBI may modify important TGFβ-related effects on arterial EC homeostasis that should be further studied in a model of inflammatory atherosclerosis.

## Supporting Information

Figure S1 **Negative control staining are presented for Ki67, lectin, von Willebrand factor (vWF) and α-smooth muscle actin (αSMA), left panel and their positive control, right panel. (Scale bar = 50 µm, original magnification ×400).**
(TIF)

Figure S2 **Immunohistological staining for BAMBI in femoral artery from WT mouse two weeks after endothelial denudation. (Secondary antibody either labeled with FITC or Texas red:original Magnification ×1000).** Again, there is typical autofluorescence of the lamina elastica interna in the green channel, but BAMBI positive endothelial cells are also clearly visible in the red channel. Arrows indicate BAMBI staining of regenerating endothelial cells. Staining for BAMBI in BAMBI^−/−^ tissue was absent and could not be distinguished from negative control stainings(not shown).
(TIF)
