# Supplementary figures and images for: Accelerated Reendothelialization, Increased Neovascularization and Erythrocyte Extravasation after Arterial Injury in BAMBI−/− Mice
Source: PLoS One. 2013 Mar 1;8(3):e58550. doi: 10.1371/journal.pone.0058550 (PMC3585719; doi:10.1371/journal.pone.0058550)

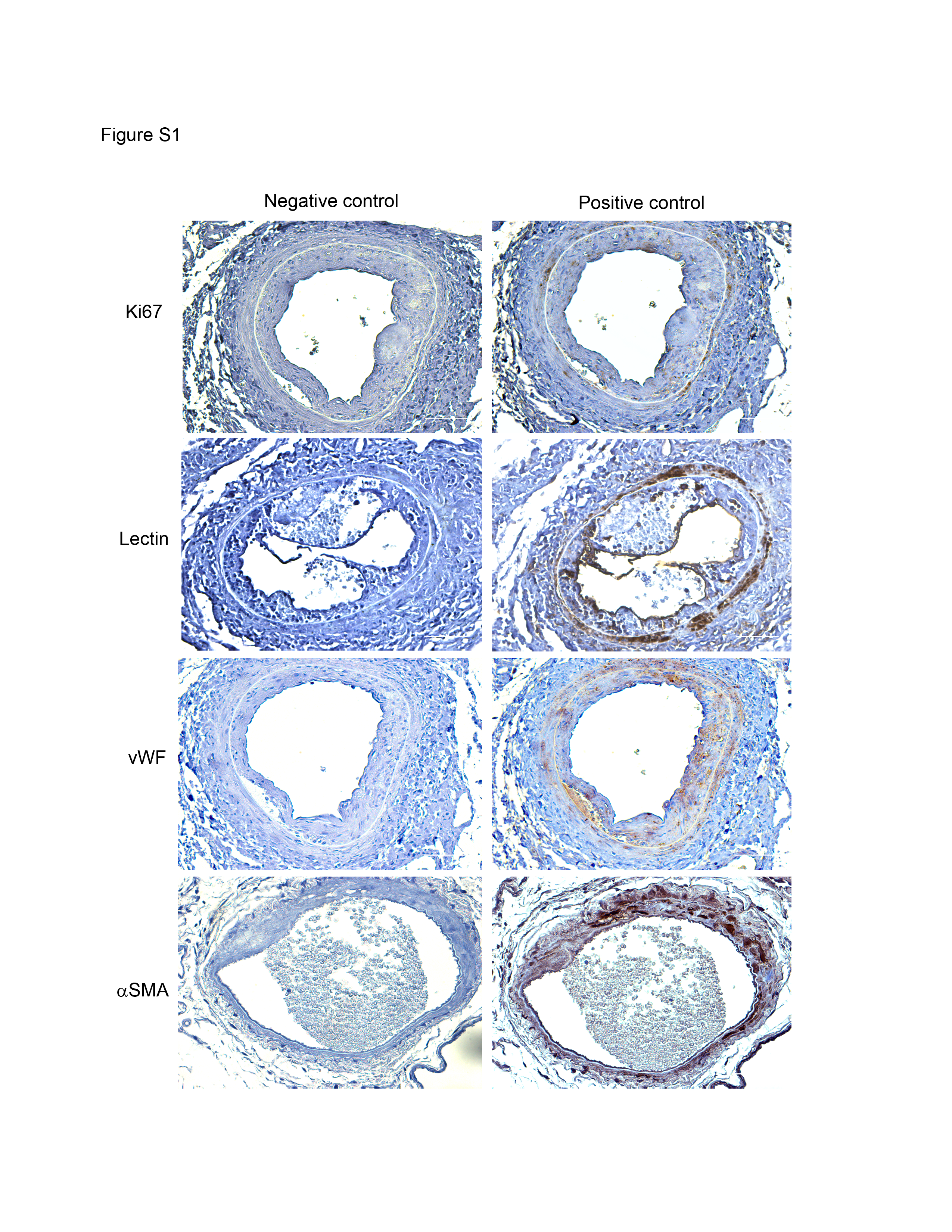

Supplement: Figure S1 — Negative control staining are presented for Ki67, lectin, von Willebrand factor (vWF) and α-smooth muscle actin (αSMA), left panel and their positive control, right panel. (Scale bar = 50 µm, original magnification ×400). (TIF) [file pone.0058550.s001.tif]

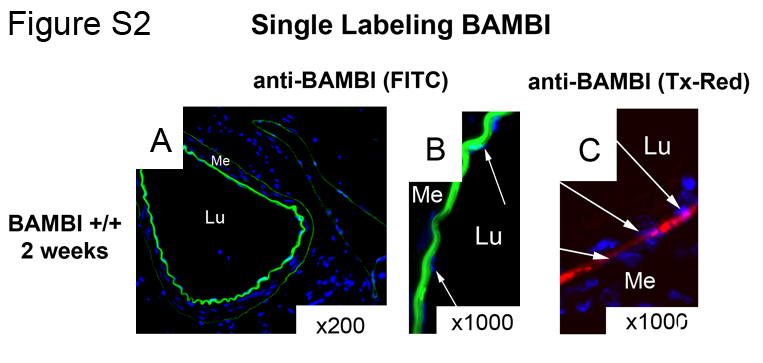

Supplement: Figure S2 — Immunohistological staining for BAMBI in femoral artery from WT mouse two weeks after endothelial denudation. (Secondary antibody either labeled with FITC or Texas red:original Magnification ×1000). Again, there is typical autofluorescence of the lamina elastica interna in the green channel, but BAMBI positive endothelial cells are also clearly visible in the red channel. Arrows indicate BAMBI staining of regenerating endothelial cells. Staining for BAMBI in BAMBI−/− tissue was absent and could not be distinguished from negative control stainings(not shown). (TIF) [file pone.0058550.s002.tif]
